# Expression of the *POTE* gene family in human ovarian cancer

**DOI:** 10.1038/s41598-018-35567-1

**Published:** 2018-11-20

**Authors:** Carter J Barger, Wa Zhang, Ashok Sharma, Linda Chee, Smitha R. James, Christina N. Kufel, Austin Miller, Jane Meza, Ronny Drapkin, Kunle Odunsi, David Klinkebiel, Adam R. Karpf

**Affiliations:** 1 Eppley Institute for Cancer Research, University of Nebraska Medical Center, Omaha, NE 68198-6805 USA; 2 Fred & Pamela Buffett Cancer Center, University of Nebraska Medical Center, Omaha, NE 68198-6805 USA; 3 Department of Pharmacology and Therapeutics, Roswell Park Comprehensive Cancer Center, Buffalo, NY 14263 USA; 4 Department of Biostatistics, Roswell Park Comprehensive Cancer Center, Buffalo, NY 14263 USA; 5 Department of Biostatistics, University of Nebraska Medical Center, Omaha, NE 68198-4375 USA; 6 Penn Ovarian Cancer Research Center, University of Pennsylvania Perelman School of Medicine, Philadelphia, PA 19104 USA; 7 Department of Immunology, Roswell Park Comprehensive Cancer Center, Buffalo, NY 14263 USA; 8 Department of Gynecologic Oncology, Roswell Park Comprehensive Cancer Center, Buffalo, NY 14263 USA; 9 Center for Immunotherapy, Roswell Park Comprehensive Cancer Center, Buffalo, NY 14263 USA; 10 Department of Biochemistry, University of Nebraska Medical Center, Omaha, NE 68198 USA

## Abstract

The *POTE* family includes 14 genes in three phylogenetic groups. We determined *POTE* mRNA expression in normal tissues, epithelial ovarian and high-grade serous ovarian cancer (EOC, HGSC), and pan-cancer, and determined the relationship of *POTE* expression to ovarian cancer clinicopathology. Groups 1 & 2 *POTEs* showed testis-specific expression in normal tissues, consistent with assignment as cancer-testis antigens (CTAs), while Group 3 *POTEs* were expressed in several normal tissues, indicating they are not CTAs. *Pan-POTE* and individual *POTE*s showed significantly elevated expression in EOC and HGSC compared to normal controls. *Pan-POTE* correlated with increased stage, grade, and the HGSC subtype. Select individual *POTEs* showed increased expression in recurrent HGSC, and *POTEE* specifically associated with reduced HGSC OS. Consistent with tumors, EOC cell lines had significantly elevated *Pan-POTE* compared to OSE and FTE cells. Notably, Group 1 & 2 *POTEs* (*POTEs A/B/B2/C/D*), Group 3 *POTE-actin* genes (*POTEs E/F/I/J/KP*), and other Group 3 *POTEs* (*POTEs G/H/M*) show within-group correlated expression, and pan-cancer analyses of tumors and cell lines confirmed this relationship. Based on their restricted expression in normal tissues and increased expression and association with poor prognosis in ovarian cancer, POTEs are potential oncogenes and therapeutic targets in this malignancy.

## Introduction

Epithelial ovarian cancer (EOC) is the most lethal gynecologic malignancy, and high-grade serous cancer (HGSC) is the most prevalent EOC subtype^1,2^. The majority of HGSC cases are diagnosed at late clinical stages. Once diagnosed, EOC and HGSC treatment consists of primary debulking surgery and platinum/taxane combination chemotherapy, typically leading to a robust clinical response. Unfortunately, most patients diagnosed in late stage ultimately relapse with chemoresistant disease^3^. Although there has been significant recent progress in ovarian cancer treatment^4–6^, there remains an urgent need for improved therapeutic approaches, particularly in the recurrent disease setting.

Cancer-testis antigens (CTAs), also known as cancer-germline genes, show low expression in normal somatic tissues but are expressed in germ cells of the adult testis and fetal ovary, and in placenta^7,8^. CTAs can show highly elevated expression in cancer, which appears most often to result from epigenetic alterations, particularly DNA hypomethylation^9–11^. Some CTAs are immunogenic (hence the name), in part because their normal expression is restricted to immune privileged sites. The immunogenicity of specific CTAs has led to the development of immunotherapies to target them in cancer, using vaccines and adoptive cell therapies^8,9^. Importantly, specific CTAs directly promote oncogenic phenotypes, suggesting they are not just cancer passengers^12–14^. This opens up new opportunities for therapeutic targeting of CTAs unrelated to immunotherapy, which is a crucial development, as only a limited number of CTAs are likely to be immunogenic.

A sizable number of CTAs, including the most frequently studied members of this superfamily, are located on the X-chromosome (CT-X genes). However, most CTAs were recently shown to be encoded on autosomes^13,15^. Amongst these, *POTEs* are the only multigene family described to date, POTEs consist of 14 primate-specific genes distributed on seven chromosomes, and are divided into three phylogenetic groups^16–18^. The *POTE* family originated from an ancestral *ankyrin repeat domain 26 (ANKRD26)* gene^17^. *POTE*s contain a conserved 3′UTR *LINE-1* element, which promoted *POTE* dispersal in the primate genome, and several Chr. 2 *POTEs* contain a C-terminal in-frame fusion with *Actin* resulting from transposition^16,19^ (Table 1). Structurally, POTE proteins contain a N-terminal cysteine-rich region, central ankyrin repeats, and C-terminal spectrin-like α-helices, suggesting participation in protein-protein interactions and association with cell membranes^19,20^.

**Table 1:** Human POTE Gene Family.

HUGO name	Original name^a^	Group^b^	Actin fusion	Testis-specific^c^
*POTEA*	*POTE8*	1	—	**√**
*POTEB*	*POTE15*	2	—	**√**
*POTEB2*	*n/a*	2	—	**√**
*POTEB3* ^d^	*n/a*	2	—	n/d
*POTEC*	*POTE18*	2	—	**√**
*POTED*	*POTE21*	2	—	**√**
*POTEE*	*POTE2γ*	3	**√**	—
*POTEF*	*POTE2α*	3	**√**	—
*POTEG*	*POTE14α*	3	—	—
*POTEH*	*POTE22*	3	—	—
*POTEI*	*POTE2β'*	3	**√**	—
*POTEJ*	*POTE2β*	3	**√**	—
*POTEKP*	*POTE2δ*	3	**√**	—
*POTEM*	*POTE14β*	3	—	—

n/a: not applicable; n/d: not determined.

^a^Corresponds to chromosomal location; Bera *et al*., *PNAS*, 2002.

^b^Based on phylogeny; Hahn *et al*., *Gene*, 2006, 238–245.

^c^GTEx RNAseq data; http://www.genecards.org/; see Supplementary Fig. S1.

^d^Excluded from mRNA expression analyses due to insufficient data.

An important early study of *POTE* expression in cancer showed differential *POTE* expression in cancer tissues, including ovarian cancer. However, the analysis of ovarian cancer was limited to an endpoint RT-PCR study of five ovarian cancer samples of unknown classification^21^. A limitation to early studies of *POTEs* was that the high homology of *POTE*s made it difficult to resolve expression of individual *POTEs*. However, in recent years, the field has experienced the advent of RNA-sequencing (RNA-seq), which can readily resolve individual *POTEs*, as well as great progress by consortia-based projects for depositing extensive RNA-seq data from normal human tissues, human tumors, and human cancer cell lines^2,22–24^. These data allow the opportunity to measure *POTE* expression in different contexts, including ovarian cancer. Here we report several new and extensive analyses of *POTE* expression, including in normal tissues, ovarian cancer tumors and cell lines, normal control cells, and an initial study in pan-cancer tissues and cell lines.

## Results

### *POTE* expression in normal human tissues

We first analyzed expression of 13/14 members of the *POTE* gene family (data was not available for *POTEB3*) (Table 1), using GTEx RNAseq data^22^, primarily to determine if *POTEs* show a testis-specific or testis-enriched expression characteristic of CTAs^7^. Notably, Groups 1 & 2 *POTEs*, which are more closely related to the ancestral *ANKRD26* gene^17^, displayed testis-specific expression (Supplementary Fig. S1), despite the fact that *ANKRD26* was widely expressed in normal tissues (data not shown). In contrast to Group 1 & 2 *POTEs*, Group 3, and particularly the *POTE-actin* genes, showed widespread normal tissue expression (Supplementary Fig. S1). The only exception was *POTEH*, a Group 3 *POTE* that showed significant expression only in testis and prostate. We conclude that Groups 1 & 2 *POTE*s (*A*, *B*, *B2*, *C*, *D*) have normal tissue expression consistent with CTAs, while Group 3 *POTEs* (*E*, *F*, *G*, *H*, *I*, *J*, *KP*, *M*) do not (Table 1). Widespread expression of *POTE-actin* genes suggests a function in normal tissues.

### *POTE* expression in EOC

**Figure 1:**
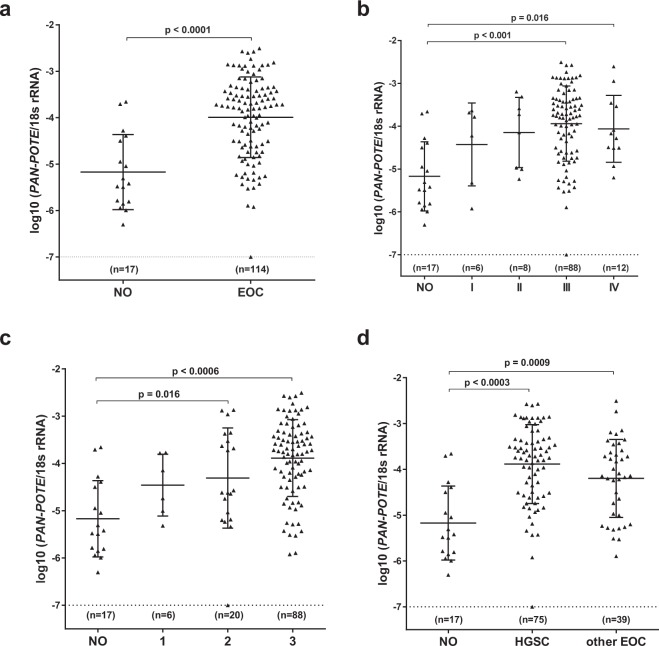
*Pan-POTE* expression in NO and EOC tissues. (**a**) NO and EOC. (**b**) NO and EOC separated by stage. (**c**) NO and EOC separated by grade. (**d**) NO and EOC separated into HGSC (serous histology, grade 2/3) and other EOC. Graphs show median values, and two-tailed Mann-Whitney tests with significant differences, after performing Bonferroni correction, are shown. Samples with no detectable *Pan-POTE* expression were plotted at log10 (−7) for clarity.

We measured *Pan-POTE* expression by RT-qPCR in EOC and bulk normal ovary (NO) tissues. Supplementary Table S1 lists the characteristics of the EOC samples. *Pan-POTE* was significantly overexpressed in EOC compared to NO, with approximately one-third of cases showing >10-fold increased expression (Fig. 1a). *Pan-POTE* expression significantly associated with increased clinical stage and pathological grade (Fig. 1b,c). We separated EOC into HGSC (serous histology, grade 2/3) and other EOC. While *Pan-POTE* was elevated in both groups compared to NO, HGSC showed significantly higher expression (Fig. 1d**)**. Individual histological subgroups did not contain sufficient samples to make meaningful comparisons (Supplementary Fig. S2**)**. Next, to assess individual *POTE* gene expression in EOC, we used Affymetrix microarrays to examine EOC (n = 40) and NO (n = 3). In agreement with *Pan-POTE* data, sub-sets of *POTE*s showed elevated expression in EOC (Supplementary Fig. S3). However, this methodology was limited by extensive *POTE* gene overlap.

### *POTE* expression in HGSC

**Figure 2:**
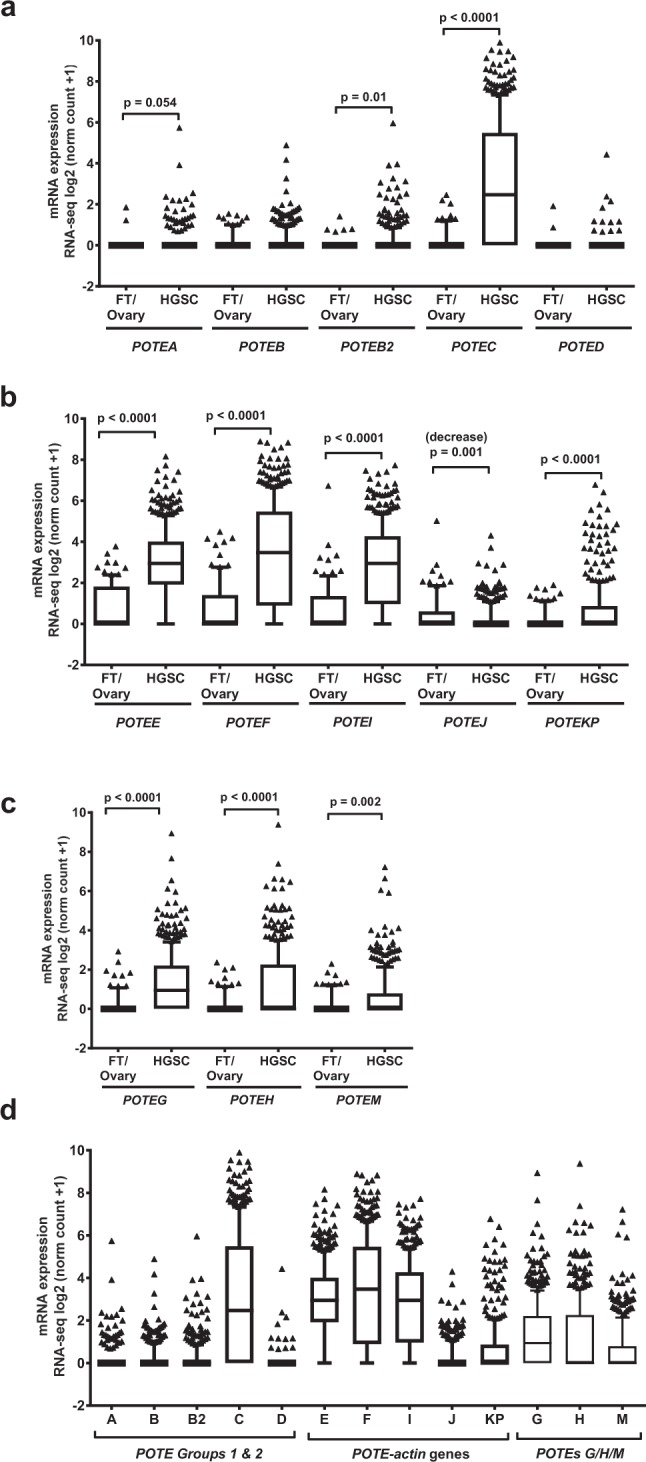
*POTE* expression in fallopian tube (FT) + ovary and HGSC. (**a**–**c**) Comparisons of *POTE* expression in normal controls (n = 93) vs. HGSC (n = 419). (**a**) Groups 1 & 2 *POTEs*. (**b**) Group 3 *POTE*-*actin* genes. (**c**) Other Group 3 *POTEs* (i.e. *POTEs G/H/M*). (**d**) Comparison of *POTE* gene expression in HGSC. Box and whiskers plot, with medians, 10–90%iles, and ranges indicated. Two-tailed Mann-Whitney tests with significant differences are shown.

HGSC frequently originates from precursor lesions in the fallopian tube epithelia (FTE), and the TCGA ovarian cancer project specifically focused on HGSC^2,25,26^. To focus our studies of *POTEs* on HGSC, and to examine individual *POTE*s using RNA-seq, we used Toil analyses^23^. As a control for HGSC, we combined normal tissue GTEx data from both ovary and fallopian tube (FT), as utilization of FT alone was not feasible due to limited sample size (n = 5), and because unseparated FT is only an approximation of FTE. This analysis revealed significant overexpression of 10/13 *POTEs* in HGSC (Fig. 2a–c). Amongst Groups 1 & 2 *POTEs*, *A*, *B2*, and *C* showed significant upregulation, along with generally low or absent expression in control tissues (Fig. 2a). All Group 3 *POTE-actin* genes showed altered expression in HGSC, with all but one (*POTEJ*) being upregulated (Fig. 2b). We noted that *POTE-actin* expression was significantly upregulated in HGSC despite expression in the control tissues. Other Group 3 *POTEs* (*POTEs G/H/M*) were also highly upregulated in HGSC, but showed lower expression in control tissues than *POTE-actin* genes (Fig. 2c). Comparison of the expression of all *POTEs* revealed that *POTEs C*, *E*, *F*, and *I* show highest overall expression in HGSC (Fig. 2d).

**Figure 3:**
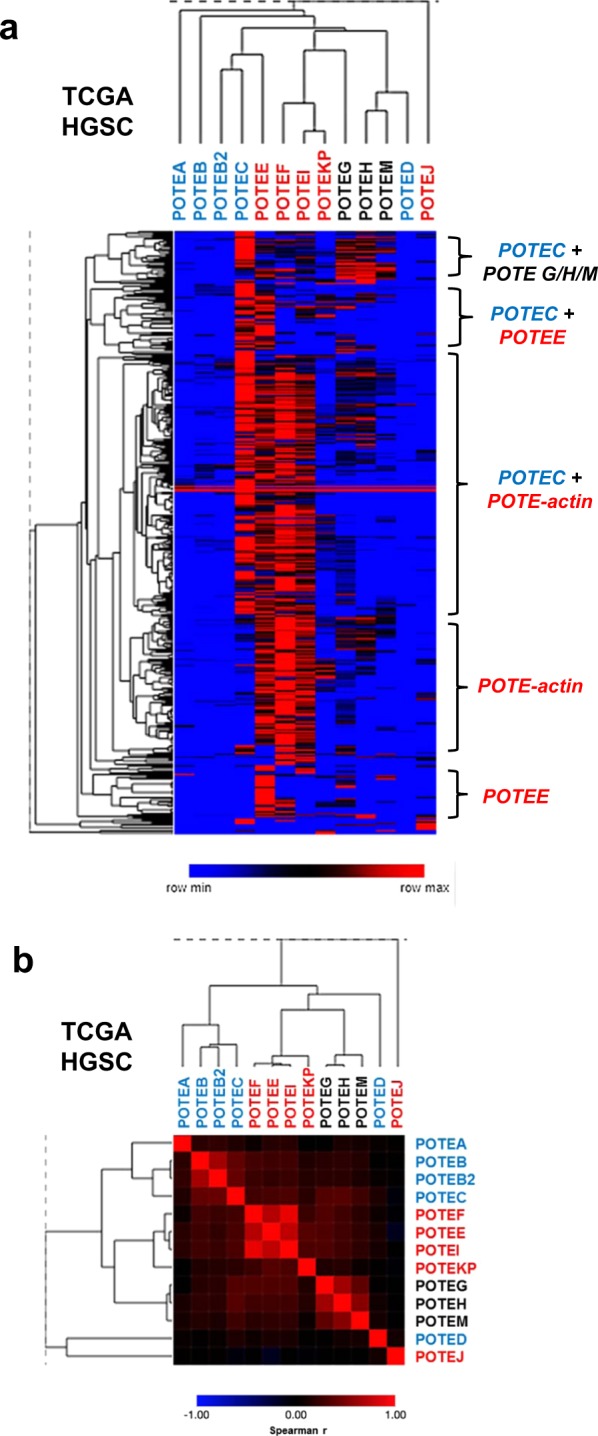
*POTE* expression in HGSC. (**a**) Expression heatmap of *POTE*s in TCGA HGSC data. Toil log2 normalized read counts shown, and coloring indicates row min to row max (see key). Samples showing enrichment for specific *POTE* expression patterns are labelled at right. (**b**) Spearman rank correlation matrix heatmap of *POTE* gene expression in TCGA HGSC. In both panels, *POTE* font color indicates *POTE* group: Groups 1 & 2 (blue), Group 3 *POTE-actin* (red), *POTE G/H/M* (black).

We used unsupervised hierarchical clustering to compare *POTE* expression in TCGA HGSC data. *POTEs* generally clustered into three expression sub-groups: i) Groups 1 & 2, ii) Group 3 *POTE-actin* genes, and iii) *POTEs G/H/M* (Fig. 3a). We also identified different tumor clusters characterized by specific *POTE* expression patterns (Fig. 3a, right labels), and the most prominent clusters were characterized by high expression of *POTEC* and/or *POTE-actin* genes. We conducted Spearman rank correlation testing of *POTE* expression, which confirmed that the three aforementioned *POTE* subgroups show correlated expression (Fig. 3b). In agreement with earlier data, the two *POTEs* that did not correlate within their respective subgroups (*POTEs D* and *J*) either showed very low expression in HGSC or were downregulated in HGSC compared to normal controls (Fig. 2a,b).

**Figure 4:**
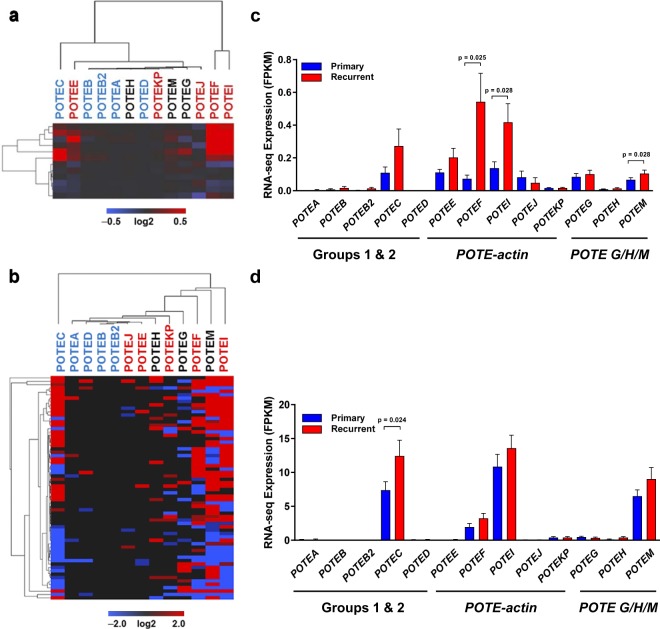
*POTE* expression in patient-matched primary and recurrent HGSC. (**a**,**b**) Expression heatmaps showing log2 fold changes for recurrent/primary HGSC. (**c**,**d**) *POTE* expression averages. (**a**,**c**) Data from Patch *et al*.^27^ (n = 12 pairs). (**b**,**d**) Data from Kreuzinger *et al*.^28^ (n = 66 pairs). Font color indicates *POTE* group: Groups 1 & 2 (blue), Group 3 *POTE-actin* (red), other Group 3 (black). (**c**) Data from^27^ (n = 12 pairs). (**d**) Data from^28^ (n = 66 pairs). Bars plot means + SEM; two-tailed student’s t-test with significant differences are shown. In panels (**a**,**b)** *POTE* font color indicates *POTE* group: Groups 1 and 2 (blue), Group 3 *POTE-actin* (red), *POTE G/H/M* (black).

HGSC patients often develop recurrent chemoresistant disease^3^. We compared *POTE* expression in patient-matched primary and recurrent HGSC using two independent RNA-seq data sets^27,28^. Data from Patch *et al*., showed altered expression of several *POTEs*, and identified *POTEF*, *I*, and M with significant upregulation in recurrent HGSC, both in individual patients and overall (Fig. 4a,c). In addition, *POTEs C* and *E* were upregulated in several patients. Data from Kreuzinger revealed a similar pattern of altered *POTE* expression, with increased expression of *POTEs C*, *F*, *I*, and M in recurrent HGSC. Only *POTEC* was significantly upregulated over the entire patient population (Fig. 4b,d). Upregulated *POTEs* included at least one member of each previously identified *POTE* expression subgroup (i.e. Groups 1 & 2, *POTE-actin* genes, and *POTE G*, *H*, *M*).

### *POTE* expression and overall survival (OS) in EOC and HGSC

**Figure 5:**
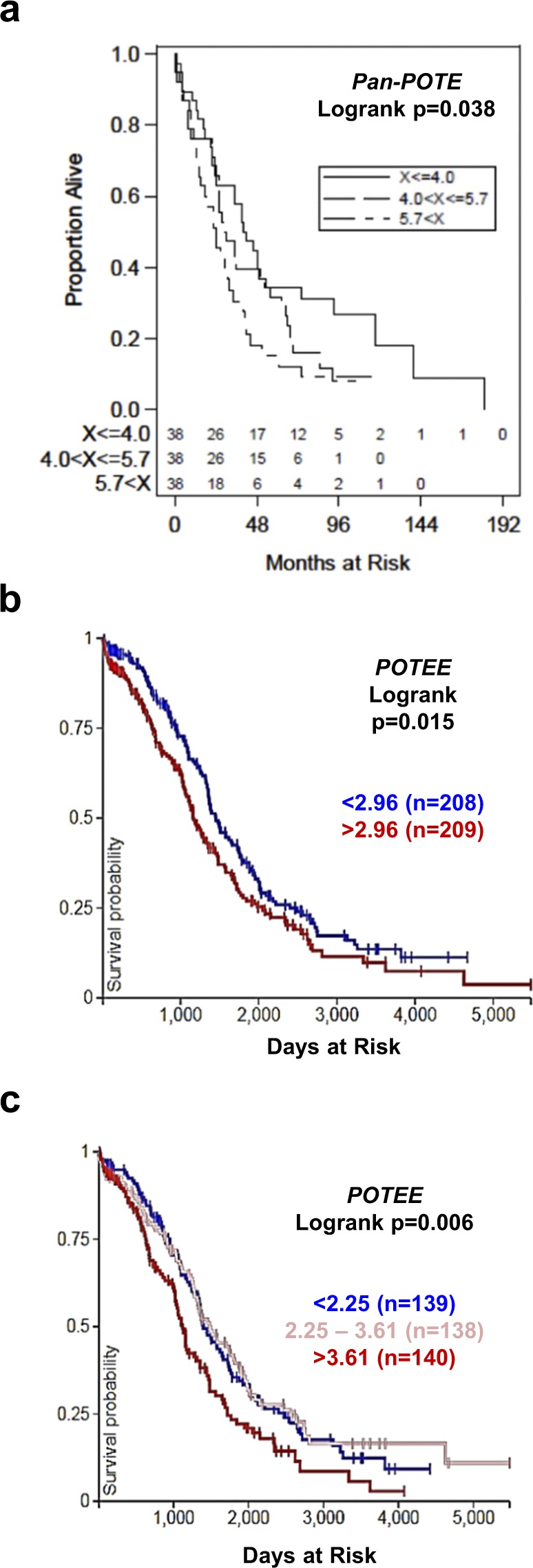
*POTE* expression and overall survival (OS) in EOC and HGSC. (**a**) *Pan-POTE* expression and OS in EOC (n = 114). (**b**,**c**) *POTEE* expression and OS in TCGA HGSC (n = 417), using either two (**b**) or three (**c**) expression subgroups.

We tested the association of *Pan-POTE* expression with OS in EOC. Consistent with the observed increase of *Pan-POTE* with stage, grade, and HGSC (Fig. 1b–d), *Pan-POTE* associated with reduced OS in a univariate analysis, but not in a multivariate analysis (Fig. 5a; data not shown). We next tested the association of individual *POTE*s with OS using HGSC TCGA data, and observed that *POTEE* associated with reduced OS, using either two or three expression sub-groups (Fig. 5b,c). Consistently, *POTEE* was upregulated in HGSC compared to normal controls, showed heterogeneous expression in HGSC, and select patients showed increased *POTEE* expression at recurrence (Figs 2b, 3a, 4a,b). Other *POTEs* were not associated with HGSC OS (data not shown).

### *POTE* expression in ovarian cancer cell lines

**Figure 6:**
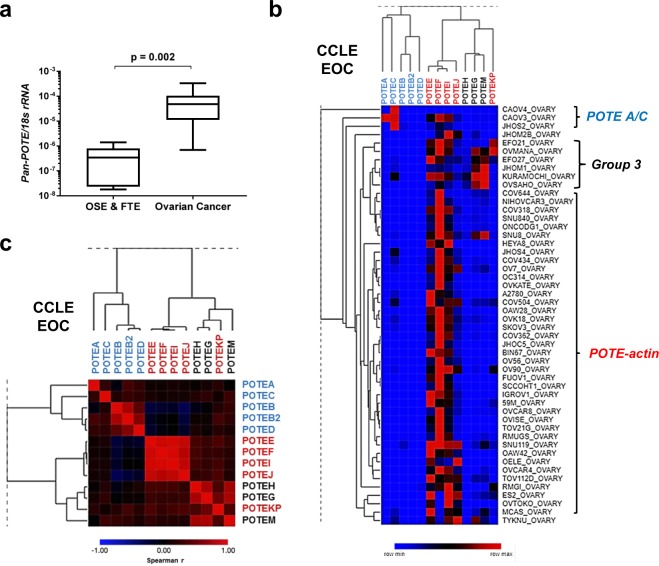
*POTE* expression in ovarian cancer and control cell lines. (**a**) *Pan-POTE* expression in control cells (ovarian surface epithelia, OSE; fallopian tube epithelia; FTE) and ovarian cancer cell lines. See Supplementary Table S1 for list of cell lines utiized. Box and whiskers plot, with medians, 25–75%iles, and ranges indicated. Two-tailed Mann-Whitney test result shown. (**b**) *POTE* expression RNA-seq read counts in CCLE ovarian cancer cell lines (n = 50). Cell line names are shown, and samples showing enrichment for specific *POTE* expression patterns are labelled at right. (**c**) Spearman rank correlation matrix heatmap of *POTE* gene expression in CCLE ovarian cancer cell lines. In panels (**b**,**c)** *POTE* font color indicates *POTE* group: Groups 1 & 2 (blue), Group 3 *POTE-actin* (red), *POTE G/H/M* (black).

Cancer cell lines are valuable tools for functional studies^29^. We measured *Pan-POTE* in a panel of cell lines relevant to EOC and HGSC, including cancer cell lines, and normal and immortalized ovarian surface epithelia (OSE) and FTE cells (Supplementary Table S2). Consistent with primary tumor data, *Pan-POTE* expression was significantly increased in ovarian cancer cells compared to control cells (Fig. 6a). Next, we examined the pattern of expression of individual *POTE*s in a large panel of ovarian cancer cell lines, using data from the cancer cell line encyclopedia (CCLE)^24^. *POTE* expression in CCLE ovarian lines segregated into the three *POTE* sub-groups described above (Fig. 6b,c). A large proportion of cell lines had elevated expression of *POTE-actin* genes (Fig. 6b).

### *POTE* expression in pan-cancer TCGA and CCLE data

**Figure 7:**
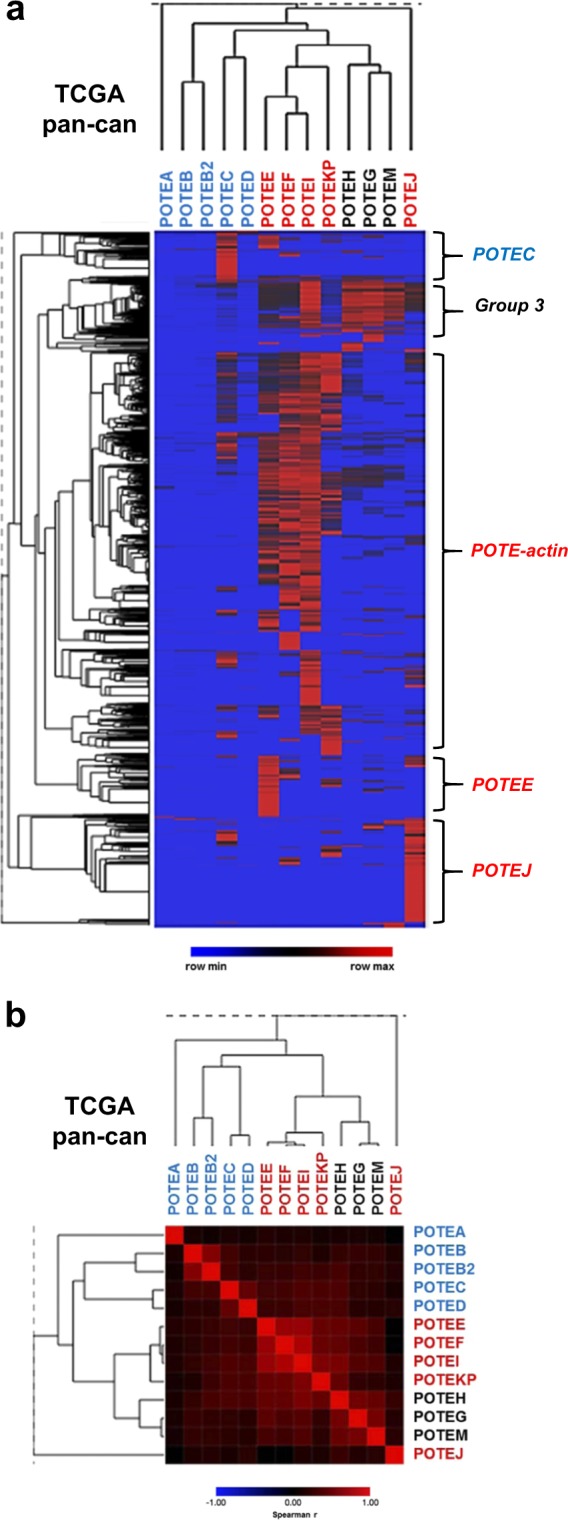
*POTE* expression in TCGA pan-cancer tissues (n = 9345), determined using RNA-seq data from the UCSC Xena browser Toil. (**a**) Unsupervised hierarchical clustering of individual *POTEs* and pan-cancer cases. log2 normalized read counts are shown. Samples showing enrichment for specific *POTE* expression patterns are labelled at right. (**b**) Spearman rank correlation matrix heatmap of *POTE* gene expression in TCGA pan-cancer data. *POTE* font color indicates *POTE* group: Groups 1 & 2 (blue), Group 3 *POTE-actin* (red), *POTE G/H/M* (black).

**Figure 8:**
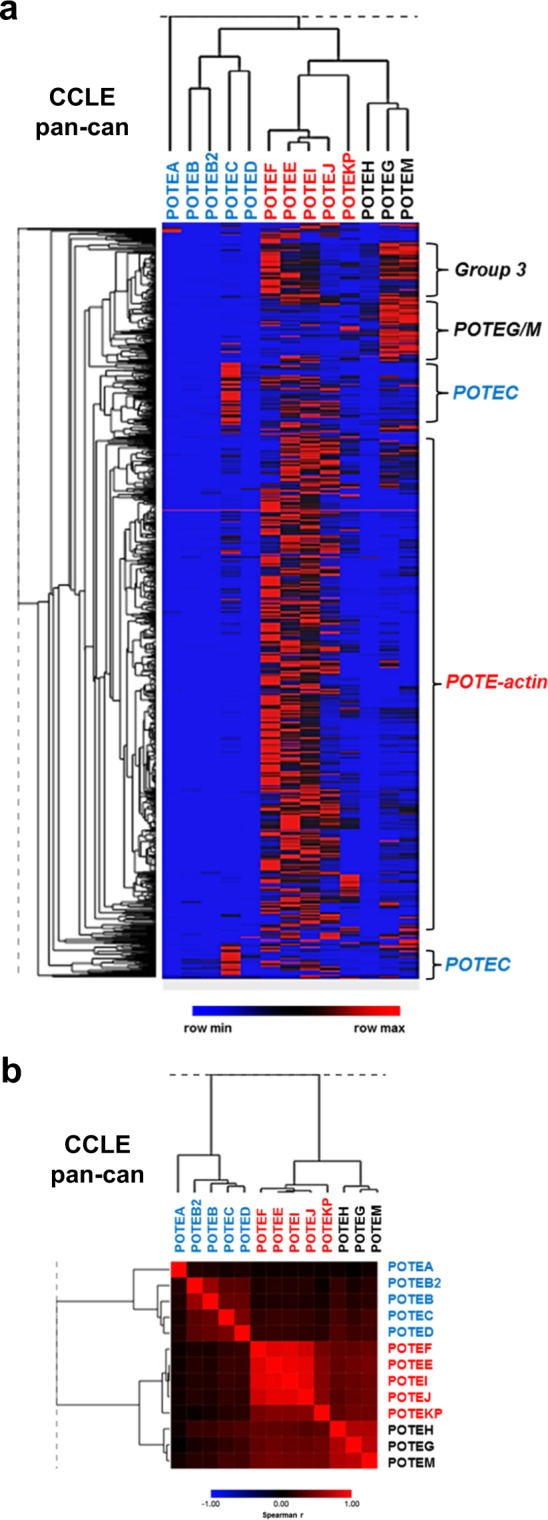
*POTE* expression in Cancer Cell Line Encyclopedia (CCLE) pan-cancer data. (**a**) *POTE* expression read counts in CCLE pan-cancer cell lines (n = 1076). Samples showing enrichment for specific *POTE* expression patterns are labelled at right. (**b**) Spearman rank correlation matrix heatmap of *POTE* gene expression in CCLE pan-cancer cell lines. *POTE* font color indicates *POTE* group: Groups 1 & 2 (blue), Group 3 *POTE-actin* (red), *POTE G/H/M* (black).

We utilized *in silico* resources to conduct an initial examination of *POTE* expression in pan-cancer^24,30^. Pan-cancer TCGA data showed similar *POTE* expression sub-groups and sample clusters as observed in HGSC (Fig. 7a,b). However, although the data were overall similar to HGSC, there were distinctions, including elevated *POTEJ* expression in a sub-set of tumors (Fig. 7a). In pan-cancer CCLE data, again similar *POTE* expression patterns were apparent, including sample clusters with increased expression of *POTEC*, *POTE-actin* genes, and Group 3 *POTEs* (Fig. 8a). Moreover, the three previously identified *POTE* expression sub-groups (Group 1 & 2, *POTE-actin* genes, *and POTE G/H/M*) perfectly segregated in pan-cancer CCLE data (Fig. 8a,b).

## Discussion

*Pan-POTE* expression is frequent in EOC and correlates with increased stage and grade, HGSC, and reduced OS. Although these data are valuable, it is important to determine individual *POTE* gene expression in the context of normal tissues and cancer. Due to extensive sequence homology this previously was difficult, requiring PCR cloning and Sanger sequencing^21^. To overcome this limitation, we utilized microarrays and, more extensively, RNA-seq. Microarray studies indicated that *POTE* sub-groups have increased expression in EOC compared to NO. Due to the availability of extensive RNA-seq data for HGSC^2^, and given our observation of *Pan-POTE* overexpression in this EOC subtype, we focused subsequent studies on HGSC. We used TCGA HGSC data, and GTEx normal FT and ovary as the control, and our analyses revealed that most individual *POTEs* (10/13 genes) are overexpressed in HGSC. Importantly, GTEx data revealed that Groups 1 & 2, but not Group 3, *POTEs* show a testis-specific expression pattern characteristic of CTAs. We conclude that Groups 1 & 2 *POTEs* are CTAs that can be overexpressed in HGSC (3/5 genes), with *POTEC* showing the most robust overexpression. In contrast, Group 3 *POTEs* are not CTAs but are more commonly overexpressed in HGSC (7/8 genes), with *POTEJ* the lone exception. A caveat to our analysis is that GTEx used bulk tissues, not specifically isolated epithelial cells^22^. Because FTE secretory cells are the progenitor cell for HGSC, future studies should determine POTE expression in this cell type, as well as in HGSC precursor lesions in the distal FT^25,26,31^. Additionally, a recent study suggests that evaluation of testis-specific expression in the context of CTA gene classification benefits from the use of isolated testicular germ cells^15^.

*POTEs* showed patterns of correlated gene expression, and the three sub-groups were: i) Groups 1 & 2 *POTEs*, ii) Group 3 *POTE-actin* genes, and iii) other Group 3 *POTEs* (i.e. *POTEs G/H/M*). These data suggest transcriptional co-regulation with sub-groups and divergence between groups. As CTA genes are regulated by epigenetic mechanisms^9^, it becomes relevant to determine whether epigenetics states, and/or specific transcription factors, explain the observed *POTE* expression sub-groups.

In addition to ovarian cancer, we conducted an initial examination of *POTE* expression in pan-cancer data sets from TCGA and CCLE. The data showed relative similarity of *POTE* expression patterns in pan-cancer. For example, sample sub-groups showed high enrichment of *POTE-actin* genes, *POTEC*, and Group 3 *POTEs*. Additionally, the three HGSC expression sub-groups were also apparent in pan-cancer data. Moving forward, it now becomes relevant to determine whether specific tumor types or lineages are enriched for specific patterns of *POTE* expression.

In contrast to *POTE* gene expression, POTE protein expression data in large cancer data sets is currently unavailable. In addition, commercial POTE antibodies recognize all or most POTEs, restricting their utility (data not shown). Supporting the relevance of our mRNA expression data, our prior studies of CTAs in EOC, including CTCFL (BORIS), CT45, and PRAME, revealed significant correlations between mRNA and protein expression^32–34^. Nevertheless, an important goal is to measure POTE protein expression levels in EOC and HGSC and to determine the relationship of protein expression to clinicopathology. Of note, a recent proteomic study reported increased POTEE expression in breast cancer^35^. It is intriguing that we observed that *POTEE* was the only *POTE* gene associated with reduced OS in HGSC, given the fact that HGSC has high genomic similarity to basal breast cancer^36^.

POTE protein expression was previously detected in human testis and spermatids, where it was associated with apoptosis^37,38^. Moreover, studies of cancer cells provide tentative support of a role for POTEs in apoptosis^39,40^. In addition, POTE-actin proteins appear likely to play a role in cytoskeletal function given their structure. Future work on POTE function in ovarian and other cancers might thus focus on apoptosis and cytoskeletal functions as starting points for investigation.

For functional cancer studies, cell lines are an invaluable tool. In this context, we observed that EOC/HGSC cell lines have significantly elevated *POTE* expression compared to normal OSE and FTE controls. In particular, POTE expression in the CCLE cell lines provides useful insight into model choice to study of POTE function in ovarian and other cancers.

The fact that several POTEs are not CTAs, combined with the high conservation of POTE proteins, could make immunological approaches to target POTEs difficult, despite the fact that POTE epitopes are capable of generating human CTL responses^41^. Regardless of the limitations in immunological targeting of POTEs, frequent POTE overexpression in EOC, HGSC, and other cancers, along with limited or absent expression in most normal tissues, supports POTEs as potential therapeutic targets. An important next step will be to determine whether (and which) POTEs have oncogenic function. Such data will provide insight into the potential of POTE-targeted approaches for cancer treatment.

## Methods

### *POTE* expression in human adult normal tissues

We determined the expression of individual *POTE*s in human adult normal tissues using GTEx^22^. We obtained GTEx RNAseq data using *GeneCards* (http://www.genecards.org/).

### *Pan-POTE* expression in human EOC and normal ovary (NO) tissues

We obtained fresh-frozen human EOC and bulk normal ovary (NO; obtained from patients without malignancy). All samples were collected using IRB-approved protocols at Roswell Park Comprehensive Cancer Center (RPCCC)^42^. All experiments using human samples were approved by the Institutional Review Board of the RPCCC and the Institutional Review Board of the University of Nebraska Medical Center (UNMC), and all methods were performed in accordance with relevant guidelines and regulations. Informed consent was obtained from all subjects and all subjects were over the age of 18. We processed tissues as described^43^. We extracted RNA using TRIzol (Invitrogen) and synthesized cDNA using iScript cDNA Synthesis Kit (BioRad). We performed qPCR using the BioRad CFX Connect system with SYBR green master mix (Qiagen), and primers from IDT. We amplified *Pan-POTE* (i.e. all *POTE* genes) as described^19^. We also determined *POTE* expression in EOC (n = 40) and NO (n = 3) using Affymetrix HG 1.0ST arrays, performed by the University at Buffalo Center of Excellence in Bioinformatics and Life Sciences (UBCOE). We normalized microarray probe cell intensity data (.cel) using the Affymetrix Expression Console (version 1.3.0.187) software running the Robust Multi-chip Averaging (RMA) background correction and quantile normalization using a linear scale.

### *POTE* expression in fallopian tube (FT), ovary, and HGSC tissues

We obtained Toil GTEx data for FT and ovary, and Toil TCGA data for HGSC and pan-cancer. All data correspond to RNA-seq normalized read counts. We obtained data from the UCSC Xena Browser (https://xenabrowser.net)^23^.

### *POTE* expression in primary and recurrent HGSC

We obtained *POTE* RNA-seq data from patient-matched primary and recurrent HGSC using the European Genome-phenome Archive (EGA) https://ega-archive.org/. We analyzed EGAD00001000877 (n = 12 pairs) and EGAD00010001403 (n = 66 pairs)^27,28^.

### *POTE* expression and overall survival (OS) in EOC and HGSC

For EOC, we defined overall survival (OS) as the time between the date of diagnosis and death, and censored patients who were alive at the time of analysis at the date of last follow up. We split EOC patients into *Pan-POTE* expression tertiles and compared OS using Kaplan-Meier analysis and Logrank test. For HGSC, we analyzed individual *POTE* expression vs. HGSC survival using the UCSC Xena Browser (https://xenabrowser.net).

### *POTE* expression in ovarian cancer, OSE, and FTE cells

We measured *Pan-POTE* expression as described above^19^. We obtained OVCAR3, A2780, and OVCAR429 from ATCC and cultured as described^43^. We obtained and cultured Kuramochi, OVSAHO, SNU119, COV318, COV362, OVCAR4, and SV40 large T-antigen immortalized normal human OSE (IOSE-SV) cells as described^44^. We obtained SKOV3 from ATCC and cultured in McCoy’s media with standard supplementation. We obtained primary human OSE from *ScienCell* and cultured according to manufacturers’ instructions. We obtained CAOV3 and OVCAR5 from Dr. Anirban Mitra and cultured as described^45^. We obtained OVCAR8 cells from the NCI and cultured in DMEM, using standard supplementation. We obtained EFO-21 from the MD Anderson Cancer Center (MDACC) Cell Line Core and cultured in RPMI 1640 and 20% FBS with standard supplementation. We obtained FU-OV1 from MDACC Cell Line Core and cultured in DMEM/F12 with standard supplementation. We obtained and cultured FT190, FT237, FT282, and FT282-CCNE1 as described^31,46,47^. We generated a clonal FT282 cell line, FT282-c11, and FT282-c11-FOXM1c cells as described in *Supplementary Methods*. We obtained CCLE RNA-seq data (normalized read counts, release date: May 2, 2018), generated and funded by Broad Cancer Dependency Map (https://depmap.org/broad/), using the Broad CCLE Portal (https://portals.broadinstitute.org/ccle/data). We analyzed data for both ovarian cancer cell lines in CCLE (**n = 50**) and pan-cancer cell lines (**n = 1076**).

### Statistical analyses

We used descriptive statistics as described in the individual figure legends to compare group differences. We used Spearman rank order tests to measure expression correlations. We assigned p < 0.05 as the cutoff for statistical significance. We used GraphPad Prism to conduct statistical analyses. Statistical analyses relevant to survival are described above.

## Electronic supplementary material

Supplementary Files

## Data Availability

The datasets generated during and/or analyzed during the current study are available from the corresponding author on reasonable request.

## References

[1] Karst AM, Drapkin R (2010). Ovarian cancer pathogenesis: a model in evolution. J Oncol.

[2] Bell D., Berchuck A., Birrer M., Chien J., Cramer D. W., Dao F., Dhir R., DiSaia P., Gabra H., Glenn P., Godwin A. K., Gross J., Hartmann L., Huang M., Huntsman D. G., Iacocca M., Imielinski M., Kalloger S., Karlan B. Y., Levine D. A., Mills G. B., Morrison C., Mutch D., Olvera N., Orsulic S., Park K., Petrelli N., Rabeno B., Rader J. S., Sikic B. I., Smith-McCune K., Sood A. K., Bowtell D., Penny R., Testa J. R., Chang K., Dinh H. H., Drummond J. A., Fowler G., Gunaratne P., Hawes A. C., Kovar C. L., Lewis L. R., Morgan M. B., Newsham I. F., Santibanez J., Reid J. G., Trevino L. R., Wu Y.-Q., Wang M., Muzny D. M., Wheeler D. A., Gibbs R. A., Getz G., Lawrence M. S., Cibulskis K., Sivachenko A. Y., Sougnez C., Voet D., Wilkinson J., Bloom T., Ardlie K., Fennell T., Baldwin J., Gabriel S., Lander E. S., Ding L., Fulton R. S., Koboldt D. C., McLellan M. D., Wylie T., Walker J., O’Laughlin M., Dooling D. J., Fulton L., Abbott R., Dees N. D., Zhang Q., Kandoth C., Wendl M., Schierding W., Shen D., Harris C. C., Schmidt H., Kalicki J., Delehaunty K. D., Fronick C. C., Demeter R., Cook L., Wallis J. W., Lin L., Magrini V. J., Hodges J. S., Eldred J. M., Smith S. M., Pohl C. S., Vandin F., Raphael B. J., Weinstock G. M., Mardis E. R., Wilson R. K., Meyerson M., Winckler W., Getz G., Verhaak R. G. W., Carter S. L., Mermel C. H., Saksena G., Nguyen H., Onofrio R. C., Lawrence M. S., Hubbard D., Gupta S., Crenshaw A., Ramos A. H., Ardlie K., Chin L., Protopopov A., Zhang Juinhua, Kim T. M., Perna I., Xiao Y., Zhang H., Ren G., Sathiamoorthy N., Park R. W., Lee E., Park P. J., Kucherlapati R., Absher D. M., Waite L., Sherlock G., Brooks J. D., Li J. Z., Xu J., Myers R. M., Laird P. W., Cope L., Herman J. G., Shen H., Weisenberger D. J., Noushmehr H., Pan F., Triche Jr T., Berman B. P., Van Den Berg D. J., Buckley J., Baylin S. B., Spellman P. T., Purdom E., Neuvial P., Bengtsson H., Jakkula L. R., Durinck S., Han J., Dorton S., Marr H., Choi Y. G., Wang V., Wang N. J., Ngai J., Conboy J. G., Parvin B., Feiler H. S., Speed T. P., Gray J. W., Levine D. A., Socci N. D., Liang Y., Taylor B. S., Schultz N., Borsu L., Lash A. E., Brennan C., Viale A., Sander C., Ladanyi M., Hoadley K. A., Meng S., Du Y., Shi Y., Li L., Turman Y. J., Zang D., Helms E. B., Balu S., Zhou X., Wu J., Topal M. D., Hayes D. N., Perou C. M., Getz G., Voet D., Saksena G., Zhang Junihua, Zhang H., Wu C. J., Shukla S., Cibulskis K., Lawrence M. S., Sivachenko A., Jing R., Park R. W., Liu Y., Park P. J., Noble M., Chin L., Carter H., Kim D., Karchin R., Spellman P. T., Purdom E., Neuvial P., Bengtsson H., Durinck S., Han J., Korkola J. E., Heiser L. M., Cho R. J., Hu Z., Parvin B., Speed T. P., Gray J. W., Schultz N., Cerami E., Taylor B. S., Olshen A., Reva B., Antipin Y., Shen R., Mankoo P., Sheridan R., Ciriello G., Chang W. K., Bernanke J. A., Borsu L., Levine D. A., Ladanyi M., Sander C., Haussler D., Benz C. C., Stuart J. M., Benz S. C., Sanborn J. Z., Vaske C. J., Zhu J., Szeto C., Scott G. K., Yau C., Hoadley K. A., Du Y., Balu S., Hayes D. N., Perou C. M., Wilkerson M. D., Zhang N., Akbani R., Baggerly K. A., Yung W. K., Mills G. B., Weinstein J. N., Penny R., Shelton T., Grimm D., Hatfield M., Morris S., Yena P., Rhodes P., Sherman M., Paulauskis J., Millis S., Kahn A., Greene J. M., Sfeir R., Jensen M. A., Chen J., Whitmore J., Alonso S., Jordan J., Chu A., Zhang Jinghui, Barker A., Compton C., Eley G., Ferguson M., Fielding P., Gerhard D. S., Myles R., Schaefer C., Mills Shaw K. R., Vaught J., Vockley J. B., Good P. J., Guyer M. S., Ozenberger B., Peterson J., Thomson E. (2011). Integrated genomic analyses of ovarian carcinoma. Nature.

[3] Bowtell DD (2015). Rethinking ovarian cancer II: reducing mortality from high-grade serous ovarian cancer. Nat Rev Cancer.

[4] Chen Y, Du H (2018). The promising PARP inhibitors in ovarian cancer therapy: From Olaparib to others. Biomed Pharmacother.

[5] Lord CJ, Ashworth A (2017). PARP inhibitors: Synthetic lethality in the clinic. Science.

[6] Konstantinopoulos, P. A. & Matulonis, U. A. PARP inhibitors in ovarian cancer: a trailblazing and transformative journey. *Clin Cancer Res*, 10.1158/1078-0432.CCR-18-1314 (2018). 10.1158/1078-0432.CCR-18-1314 29871906

[7] Simpson AJ, Caballero OL, Jungbluth A, Chen YT, Old LJ (2005). Cancer/testis antigens, gametogenesis and cancer. Nat Rev Cancer.

[8] Coulie PG, V den Eynde BJ, van der Bruggen P, Boon T (2014). Tumour antigens recognized by T lymphocytes: at the core of cancer immunotherapy. Nat Rev Cancer.

[9] Akers SN, Odunsi K, Karpf AR (2010). Regulation of cancer germline antigen gene expression: implications for cancer immunotherapy. Future Oncol.

[10] De Smet C, Loriot A (2013). DNA hypomethylation and activation of germline-specific genes in cancer. Adv Exp Med Biol.

[11] Want Muzamil Y., Lugade Amit A., Battaglia Sebastiano, Odunsi Kunle (2018). Nature of tumour rejection antigens in ovarian cancer. Immunology.

[12] Whitehurst AW (2014). Cause and consequence of cancer/testis antigen activation in cancer. Annu Rev Pharmacol Toxicol.

[13] Wang C (2016). Systematic identification of genes with a cancer-testis expression pattern in 19 cancer types. Nat Commun.

[14] Maxfield KE (2015). Comprehensive functional characterization of cancer-testis antigens defines obligate participation in multiple hallmarks of cancer. Nat Commun.

[15] Bruggeman Jan Willem, Koster Jan, Lodder Paul, Repping Sjoerd, Hamer Geert (2018). Massive expression of germ cell-specific genes is a hallmark of cancer and a potential target for novel treatment development. Oncogene.

[16] Bera TK (2002). POTE, a highly homologous gene family located on numerous chromosomes and expressed in prostate, ovary, testis, placenta, and prostate cancer. Proc Natl Acad Sci USA.

[17] Hahn Y, Bera TK, Pastan IH, Lee B (2006). Duplication and extensive remodeling shaped POTE family genes encoding proteins containing ankyrin repeat and coiled coil domains. Gene.

[18] Bera TK (2008). Selective POTE paralogs on chromosome 2 are expressed in human embryonic stem cells. Stem Cells Dev.

[19] Lee Y (2006). Evolution and expression of chimeric POTE-actin genes in the human genome. Proc Natl Acad Sci USA.

[20] Bera TK (2004). Five POTE paralogs and their splice variants are expressed in human prostate and encode proteins of different lengths. Gene.

[21] Bera TK (2006). POTE paralogs are induced and differentially expressed in many cancers. Cancer Res.

[22] Consortium GT (2013). The Genotype-Tissue Expression (GTEx) project. Nat Genet.

[23] Vivian J (2017). Toil enables reproducible, open source, big biomedical data analyses. Nat Biotechnol.

[24] Barretina J (2012). The Cancer Cell Line Encyclopedia enables predictive modelling of anticancer drug sensitivity. Nature.

[25] Klinkebiel D, Zhang W, Akers SN, Odunsi K, Karpf AR (2016). DNA Methylome Analyses Implicate Fallopian Tube Epithelia as the Origin for High-Grade Serous Ovarian Cancer. Mol Cancer Res.

[26] Labidi-Galy SI (2017). High grade serous ovarian carcinomas originate in the fallopian tube. Nat Commun.

[27] Patch AM (2015). Whole-genome characterization of chemoresistant ovarian cancer. Nature.

[28] Kreuzinger C (2017). A Complex Network of Tumor Microenvironment in Human High-Grade Serous Ovarian Cancer. Clin Cancer Res.

[29] Domcke S, Sinha R, Levine DA, Sander C, Schultz N (2013). Evaluating cell lines as tumour models by comparison of genomic profiles. Nat Commun.

[30] Liu J (2018). An Integrated TCGA Pan-Cancer Clinical Data Resource to Drive High-Quality Survival Outcome Analytics. Cell.

[31] Perets R (2013). Transformation of the fallopian tube secretory epithelium leads to high-grade serous ovarian cancer in Brca;Tp53;Pten models. Cancer Cell.

[32] Woloszynska-Read A (2011). Coordinated cancer germline antigen promoter and global DNA hypomethylation in ovarian cancer: association with the BORIS/CTCF expression ratio and advanced stage. Clin Cancer Res.

[33] Zhang W (2016). PRAME expression and promoter hypomethylation in epithelial ovarian cancer. Oncotarget.

[34] Zhang W (2015). DNA hypomethylation-mediated activation of Cancer/Testis Antigen 45 (CT45) genes is associated with disease progression and reduced survival in epithelial ovarian cancer. Epigenetics.

[35] Cine N (2014). Identification of ApoA1, HPX and POTEE genes by omic analysis in breast cancer. Oncol Rep.

[36] Cancer Genome Atlas, N. (2012). Comprehensive molecular portraits of human breast tumours. Nature.

[37] Bera TK, Walker DA, Sherins RJ, Pastan I (2012). POTE protein, a cancer-testis antigen, is highly expressed in spermatids in human testis and is associated with apoptotic cells. Biochem Biophys Res Commun.

[38] Ise T (2008). Expression of POTE protein in human testis detected by novel monoclonal antibodies. Biochem Biophys Res Commun.

[39] Redfield SM (2013). TheC-terminal common to group 3 POTES (CtG3P): a newly discovered nucleolar marker associated with malignant progression and metastasis. Am J Cancer Res.

[40] Liu XF, Bera TK, Liu LJ, Pastan I (2009). A primate-specific POTE-actin fusion protein plays a role in apoptosis. Apoptosis.

[41] Huang YH (2013). Identification and enhancement of HLA-A2.1-restricted CTL epitopes in a new human cancer antigen-POTE. PLoS One.

[42] Akers SN (2014). LINE1 and Alu repetitive element DNA methylation in tumors and white blood cells from epithelial ovarian cancer patients. Gynecol Oncol.

[43] Woloszynska-Read A (2007). DNA methylation-dependent regulation of BORIS/CTCFL expression in ovarian cancer. Cancer Immun.

[44] Barger CJ (2015). Genetic determinants of FOXM1 overexpression in epithelial ovarian cancer and functional contribution to cell cycle progression. Oncotarget.

[45] Mitra AK (2015). *In vivo* tumor growth of high-grade serous ovarian cancer cell lines. Gynecol Oncol.

[46] Karst AM, Drapkin R (2012). Primary culture and immortalization of human fallopian tube secretory epithelial cells. Nat Protoc.

[47] Karst AM (2014). Cyclin E1 deregulation occurs early in secretory cell transformation to promote formation of fallopian tube-derived high-grade serous ovarian cancers. Cancer Res.

