# Supplementary material for: Expression of the POTE gene family in human ovarian cancer
Source: Sci Rep. 2018 Nov 20;8:17136. doi: 10.1038/s41598-018-35567-1 (PMC6244393; doi:10.1038/s41598-018-35567-1)
Supplement: Supplementary file 1 — Supplementary Files [file 41598_2018_35567_MOESM1_ESM.pdf]

Supplementary Information for:

Expression of the *POTE* gene family in human ovarian cancer

Carter J Barger, Wa Zhang, Ashok Sharma, Linda Chee, Smitha R. James, Christina N. Kufel, Austin Miller, Jane Meza, Ronny Drapkin, Kunle Odunsi, David Klinkebiel, and Adam R. Karpf

Supplementary Figures

Supplementary Figure S1. *POTE* gene family expression in human adult normal tissues. We analyzed GTEx RNAseq data [1] using *GeneCards* (http://www.genecards.org/).

Supplementary Figure S2. *Pan-POTE* expression in primary NO and EOC histological subtypes.

Supplementary Figure S3. *POTE* expression in primary NO (n=3) and EOC (n=40), determined using Affymetrix HG 1.0ST microarrays. Log2 expression changes for EOC/NO are shown.

Supplementary Tables

Supplementary Table S1. EOC sample information.

Supplementary Table S2. List and description of cell lines used in Figure 6A.

Supplementary Methods

Generation of clonal FT282 cells (FT282-c11) cells. Conditioned media was made by adding fresh FTE medium (DMEM/F12, 10% FBS, 1% P/S) to a culture of FT282 cells grown to a confluency of 70-80%. After 24 hours, the conditioned media was collected from the cells and filtered through a 0.22-mm low-protein-binding filter to remove any floating cells. Single cell clones were derived from FT282 cells using sterile glass cloning cylinders (Sigma, 10 mm x 10 mm). A culture of FT282 cells were trypsinized, pelleted and resuspended in 1:1 mixture of conditioned media:fresh FTE media. Cells were counted with a hemocytometer and inspected to confirm a single cell suspension. FT282 cells were seeded into 2-15 cm dishes, 1,000 cells per dish. 1:1 mixture of conditioned media:fresh FTE media was replenished every 72 hours. After clones reached a size greater than 100 cells, 12 clones (C1-C12) were picked with glass cloning cylinders. Media was removed from the 15 cm dish and washed with PBS. Vacuum grease was applied to one side of the cloning cylinders and placed over the selected clones. 50 µl of trypsin was added to each cloning cylinder and the dish was placed at 37 ºC to trypsinze. After cells rounded up then 500 µl of FTE media was added to the cloning cylinder and the

cells were transferred to single well within a 48-well dish. Upon confluency, clonal cells were progressively passaged to larger dishes (24-well, 12-well, 6-well, 60 mm, 10 cm). Several clonal cell lines were expanded and characterized. All clones were confirmed to be derived from parental FT282 cells using the following: STR Analysis (University of Illinois at Chicago), RT-PCR for *TERT* mRNA expression, Western blot for V5 (p53-R175H), and Western blot to confirm high PAX8 and low calretinin protein expression. Cells were confirmed to be Mycoplasma negative (UNMC Epigenomics Core Facility). One clone (FT282-c11) was selected as representative for further experimentation.

Generation of FT282-c11-FOXM1c cells. The tetracycline-inducible lentiviral vector, pCW57.1-FOXM1c (Addgene #68810) was used to generate FT282-c11-FOXM1c cells [2]. Replication-deficient lentivirus expressing tetracycline-inducible FOXM1 was produced by transient transfection of 6.0 µg psPAX2 (Addgene #12260), 2.0 µg pMD2.G (Addgene #12259), and 8.0 µg transfer plasmid into HEK293T cells in a 10-cm dish with Lipofectamine 2000 reagent (Life Technologies), according to the manufacturer’s instructions. Viral supernatants were collected at 48 hours, passed through a 0.22-µm filter, and titered by serial dilution with puromycin (Life Technologies) selection and colony formation. The highest dilution producing drug selected colonies was used to transduce FT282-c11 cells in the presence of polybrene (4 µg/ml, Sigma), and 1 µg/ml puromycin was introduced 48 hours post-infection. After five days of puromycin selection, cells were allowed to recover and expand for one week. Cells were seeded in 6-well plates and the next day media was changed with or without doxycycline (Sigma) to induce transgene expression. Media with or without doxycycline was changed every 24 hours.

Supplementary References

1 Consortium, G. T. The Genotype-Tissue Expression (GTEx) project. *Nat Genet* **45**, 580-585, doi:10.1038/ng.2653 (2013).

2 Barger, C. J. *et al.* Genetic determinants of FOXM1 overexpression in epithelial ovarian cancer and functional contribution to cell cycle progression. *Oncotarget* **6**, 27613-27627, doi:10.18632/oncotarget.4546 (2015).

Supplementary Figure S1.

Group 1 & 2 *POTEs*

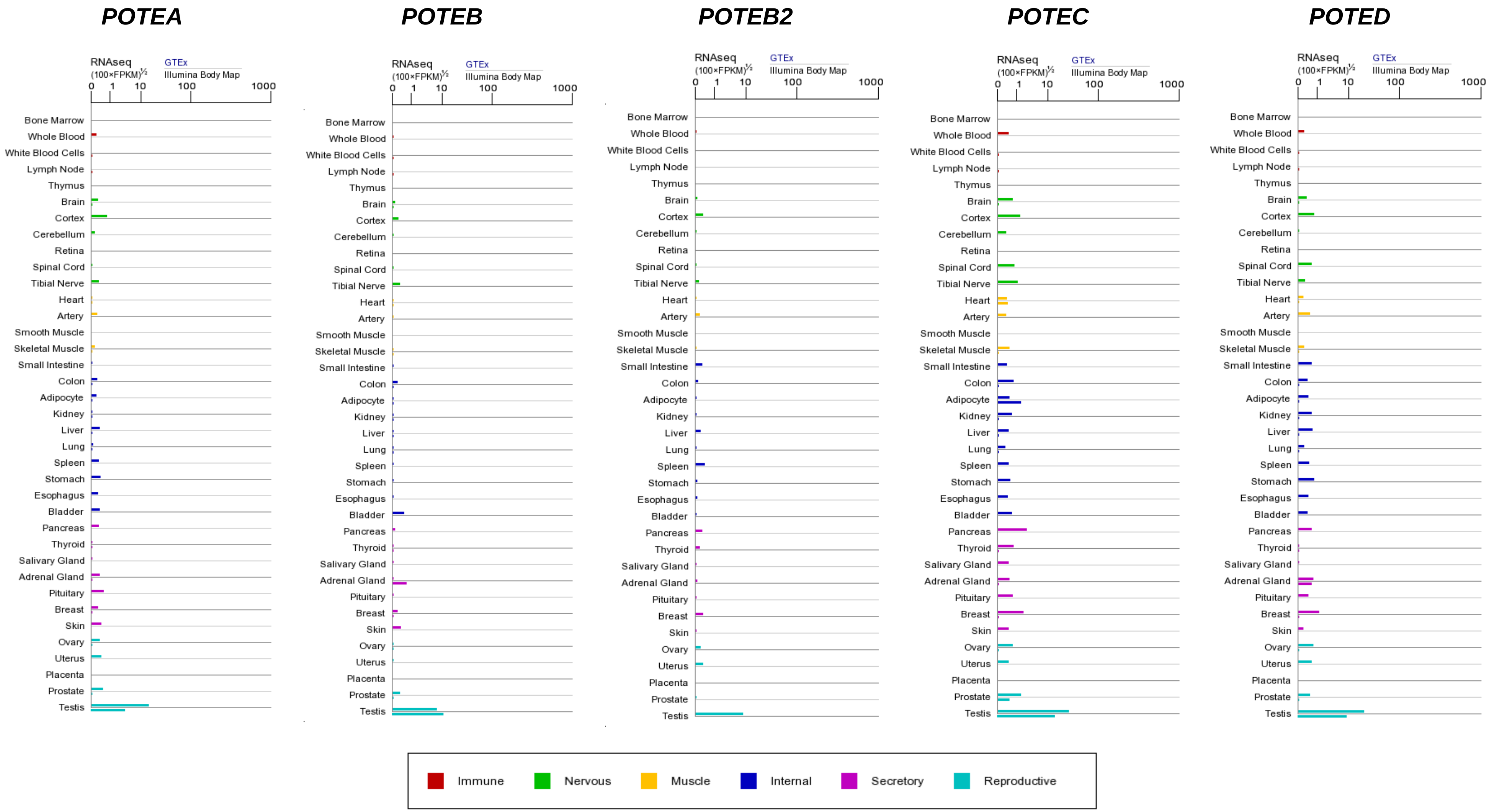

Group 3 *POTE-actin* genes

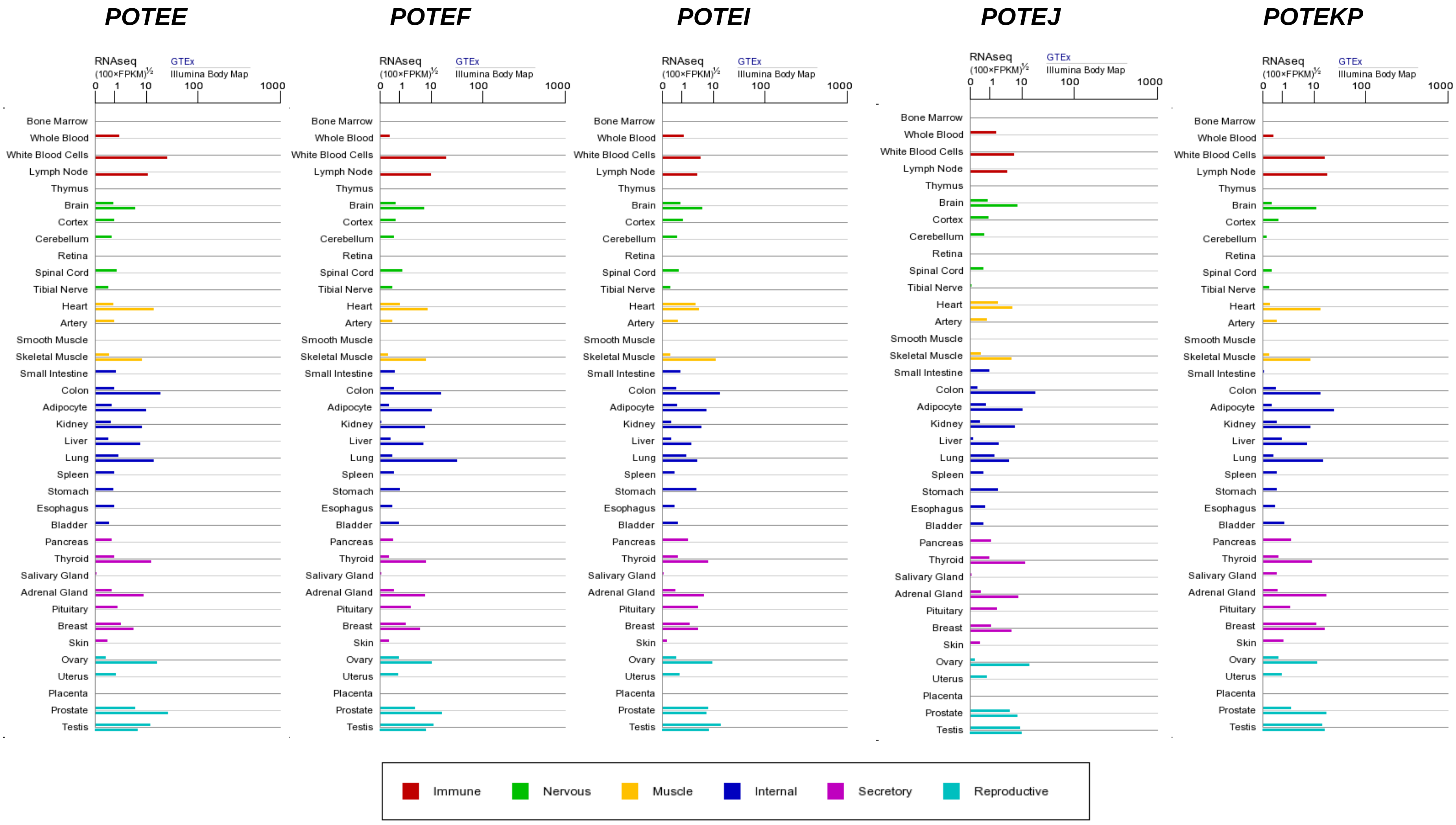

Group 3 *POTEs G/H/M*

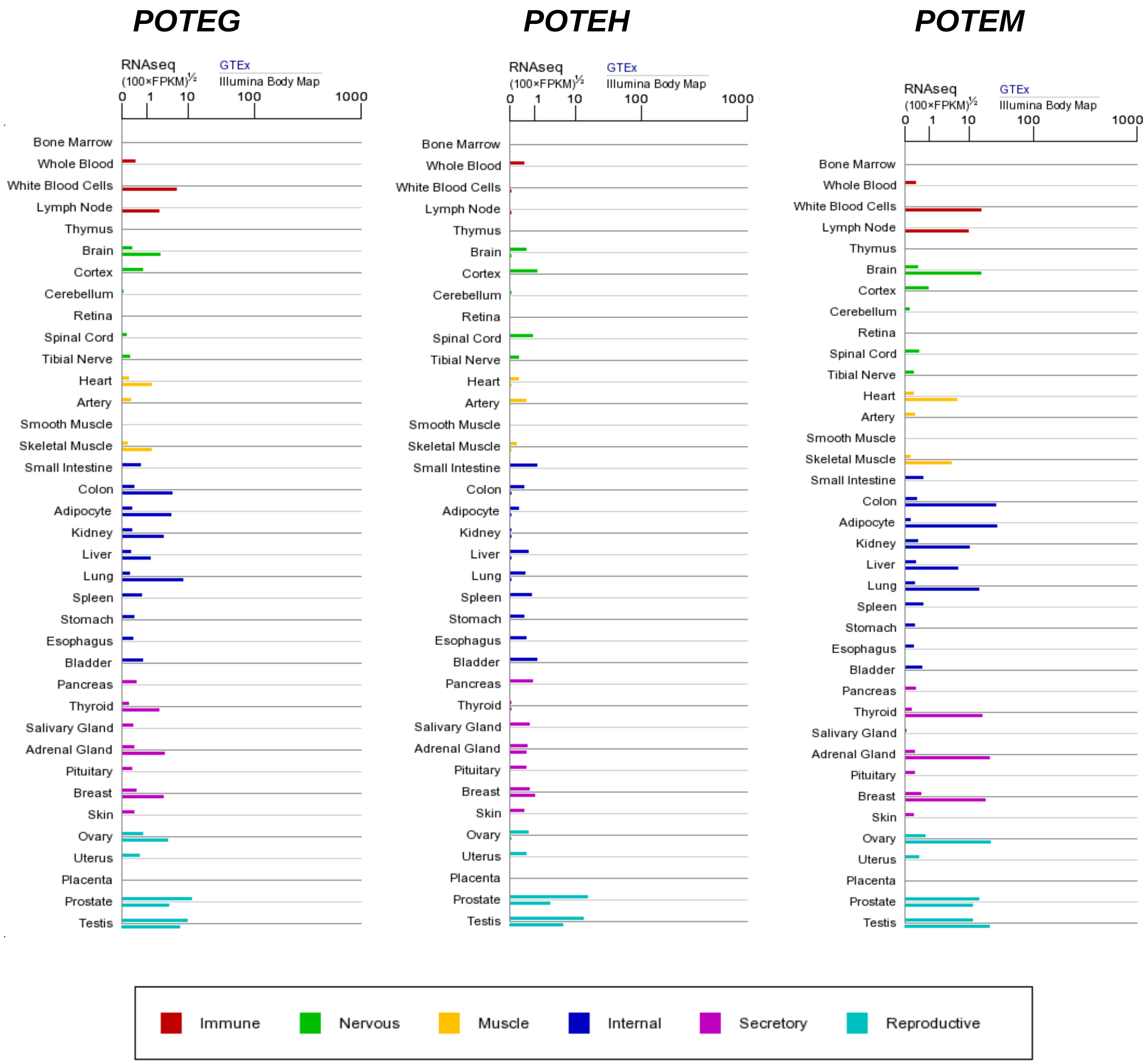

Supplementary Figure S2.

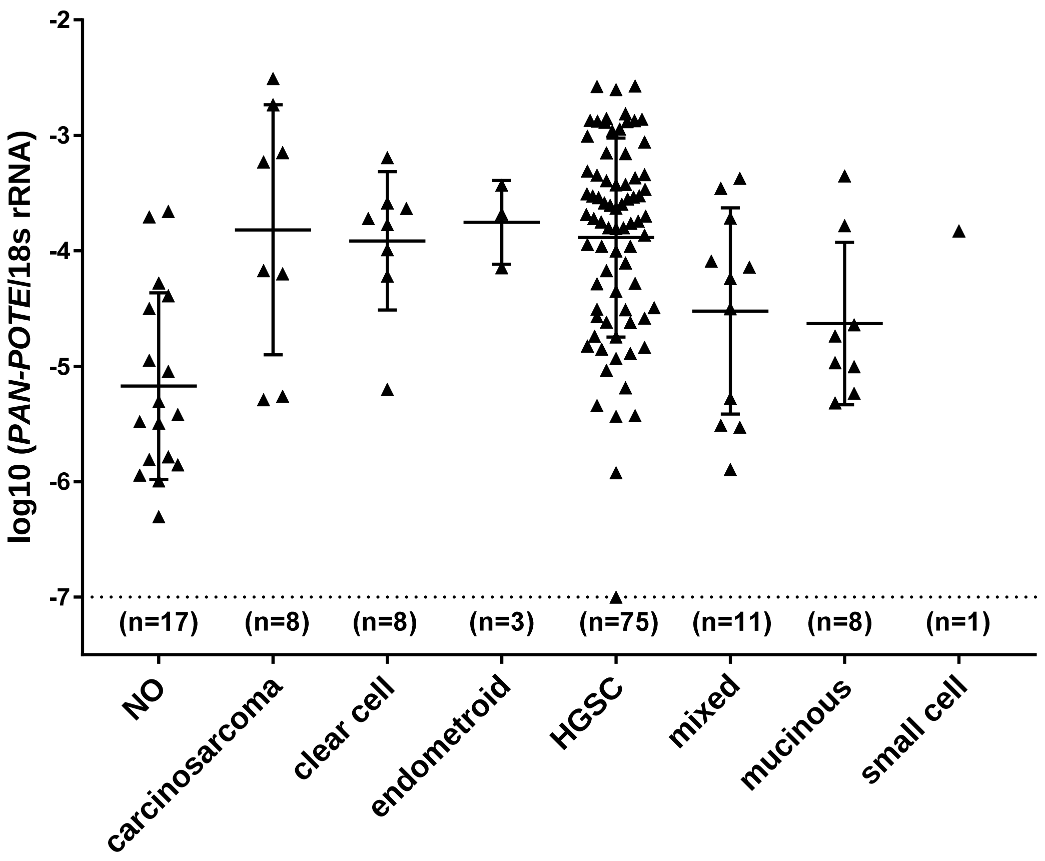

Supplementary Figure S3.

Probe ID	EOC/NO log2	Affymetrix Gene Annotation
8055153	2.34	*POTEG /// POTEM /// POTEE /// POTEB2 /// POTEB /// POTEJ /// POTEB3 /// LOC102723502*
8045257	2.14	*POTEG /// POTEM /// POTEE /// POTEB2 /// POTEB /// POTEJ /// POTEB3 /// LOC102723502*
8055222	2.01	*POTEG /// POTEM /// POTEE /// POTEB2 /// POTEB /// POTEI /// POTEJ /// POTEB3 /// LOC102723502*
7977456	1.44	*POTEG /// POTEM*
8067844	1.43	*POTEG /// POTEC /// POTED /// POTEM /// POTEB2 /// POTEB /// POTEB3 /// LOC100288966 /// LOC102723502*
8022428	1.32	*POTEG /// POTEM /// POTEC /// POTEB2 /// POTEB /// POTEB3 /// LOC102723502*
7986605	1.21	*POTEG /// POTEM /// POTEB2 /// POTEB /// POTEB3 /// LOC102723502*
8074170	1.19	*POTEG /// POTEM*
7972983	1.13	*POTEG /// POTEM*
8045208	0.48	*POTEM /// POTEJ /// POTEG*
8045321	0.35	*POTEM /// POTEJ /// POTEKP /// POTEG*
8055151	0.22	*POTEF /// POTEM /// POTEJ /// POTEG*
7977454	0.21	*POTEM /// POTEG*
8055220	0.14	*POTEM /// POTEI /// POTEJ /// POTEG*
7988281	0.10	*POTEM /// POTEG*
8113936	0.08	*POTEM /// POTEG*
8107096	0.04	*POTEM /// ACTG1P1 /// POTEG*
8146307	0.01	*POTEA*
8083032	0.01	*POTEM /// ACTG1P1 /// POTEG*
8106475	-0.03	*POTEM /// POTEG*

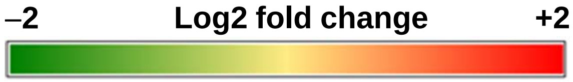

Supplementary Table S1.

Histotype	Stage	Grade	N	Histotype N (% of total)	Histotype age mean (range)
Carcinosarcoma	IIIC	3	8	8 (7.0%)	67.4 (53-89)
Clear Cell	IA	2	1		
	IC	3	1		
	IIB	2	2		
	IIC	3	1		
	IIIC	1	1		
	IIIC	3	1		
	IV	2	1	8 (7.0%)	54 (49-60)
Endometroid	IC	2	1		
	IIB	1	1		
	IIIC	3	1	3 (2.6%)	63.3 (52-73)
High Grade Serous	IC	3	1		
	IIC	3	1		
	IIIB	3	2		
	IIIC	2	13		
	IIIC	3	49		
	IV	3	9	75 (65.8%)	63.2 (22-89)
Mixed	IIIC	2	1		
	IIIC	3	8		
	IV	3	2	11 (9.7%)	66.8 (49-84)
Mucinous	IA	1	2		
	IIB	1	1		
	IIB	3	1		
	IIC	2	1		
	IIIC	1	1		
	IIIC	2	2	8 (7.0%)	62.6 (21-84)
Small Cell	IIIC	3	1	1 (0.9%)	49
Total EOC				114 (100%)	63.1 (21-89)

Supplementary Table S2. Cell lines used in Fig 6A.

Cell Type[1]	Name	Description	Source/Reference
**OSE**	**hOSE**	Primary human OSE	www.sciencellonline.com
	**IOSE-SV**	OSE immortalized with SV40 LTag	[1]
**FTE**	**FT190**	FTSEC immortalized with hTERT + SV40LTag	[2]
	**FT237**	FTSEC immortalized with hTERT + shP53 + CDK4-R24C	[3]
	**FT282-c11**	FTSEC immortalized with hTERT + p53R175H (clonal)	Current study
	**FT282-FOXM1c**	FT282-c11 with transgenic FOXM1c	Current study
	**FT282-CCNE1**	FT282 with transgenic cyclin E1	[4]
**EOC/HGSC**	**OVCAR429**	Clear cell adenocarcinoma	[5]
	**SNU119**	Likely HGSC	[6]
	**OVCAR5**	HGSC histology in xenograft	[7]
	**Kuramochi**	Likely HGSC	[6]
	**EFO-21**	Possibly HGSC	[6]
	**OVCAR4**	Likely HGSC	[6]
	**COV362**	Likely HGSC	[6]
	**OVCAR8**	Possibly HGSC	[6]
	**FU-OV1**	HGSC	[8]
	**OVCAR3**	Possibly HGSC	[6]
	**CAOV3**	Likely HGSC	[6]
	**A2780**	Resembles endometriod	[6]
	**OVSAHO**	Likely HGSC	[6]
	**COV318**	Likely HGSC	[6]

[1] OSE, ovarian surface epithelia; FTE, fallopian tube epithelia; EOC, epithelial ovarian cancer; HGSC, high-grade serous ovarian cancer

<u>References</u>

[1] Barger CJ, Zhang W, Hillman J, Stablewski AB, Higgins MJ, Vanderhyden BC, et al. Genetic determinants of FOXM1 overexpression in epithelial ovarian cancer and functional contribution to cell cycle progression. Oncotarget. 2015;6:27613-27.
[2] Perets R, Wyant GA, Muto KW, Bijron JG, Poole BB, Chin KT, et al. Transformation of the fallopian tube secretory epithelium leads to high-grade serous ovarian cancer in Brca;Tp53;Pten models. Cancer Cell. 2013;24:751-65.
[3] Karst AM, Drapkin R. Primary culture and immortalization of human fallopian tube secretory epithelial cells. Nat Protoc. 2012;7:1755-64.
[4] Karst AM, Jones PM, Vena N, Ligon AH, Liu JF, Hirsch MS, et al. Cyclin E1 deregulation occurs early in secretory cell transformation to promote formation of fallopian tube-derived high-grade serous ovarian cancers. Cancer Res. 2014;74:1141-52.
[5] Shaw TJ, Senterman MK, Dawson K, Crane CA, Vanderhyden BC. Characterization of intraperitoneal, orthotopic, and metastatic xenograft models of human ovarian cancer. Mol Ther. 2004;10:1032-42.
[6] Domcke S, Sinha R, Levine DA, Sander C, Schultz N. Evaluating cell lines as tumour models by comparison of genomic profiles. Nat Commun. 2013;4:2126.
[7] Mitra AK, Davis DA, Tomar S, Roy L, Gurler H, Xie J, et al. In vivo tumor growth of high-grade serous ovarian cancer cell lines. Gynecol Oncol. 2015;138:372-7.
[8] Emoto M, Oshima K, Ishiguro M, Iwasaki H, Kawarabayashi T, Kikuchi M. Establishment and characterization of a serous papillary adenocarcinoma cell line of the human ovary in a serum-free culture. Pathol Res Pract. 1999;195:237-42.
